# Multiple Stressors at the Land-Sea Interface: Cyanotoxins at the Land-Sea Interface in the Southern California Bight

**DOI:** 10.3390/toxins9030095

**Published:** 2017-03-09

**Authors:** Avery O. Tatters, Meredith D.A. Howard, Carey Nagoda, Lilian Busse, Alyssa G. Gellene, David A. Caron

**Affiliations:** 1 Department of Biological Sciences, University of Southern California, 3616 Trousdale Parkway, Los Angeles, CA 90089-0371, USA; gellene@usc.edu (A.G.G.); dcaron@usc.edu (D.A.C.); 2 Southern California Coastal Water Research Project, 3535 Harbor Boulevard, Suite 110, Costa Mesa, CA 92626, USA; meredithh@sccwrp.org; 3 San Diego Regional Water Quality Control Board, 2375 Northside Drive, Suite 100, San Diego, CA 92108, USA; carey.nagoda@waterboards.ca.gov; 4 German Federal Environmental Agency, Umweltbundesamt, Wörlitzer Platz 1, 06844 Dessau, Germany; lilian.busse@uba.de

**Keywords:** cyanobacteria, cyanotoxin, estuary, microcystin, anatoxin, cylindrospermopsin, nodularin, saxitoxin, Southern California Bight

## Abstract

Blooms of toxic cyanobacteria in freshwater ecosystems have received considerable attention in recent years, but their occurrence and potential importance at the land-sea interface has not been widely recognized. Here we present the results of a survey of discrete samples conducted in more than fifty brackish water sites along the coastline of southern California. Our objectives were to characterize cyanobacterial community composition and determine if specific groups of cyanotoxins (anatoxins, cylindrospermopsins, microcystins, nodularins, and saxitoxins) were present. We report the identification of numerous potentially harmful taxa and the co-occurrence of multiple toxins, previously undocumented, at several locations. Our findings reveal a potential health concern based on the range of organisms present and the widespread prevalence of recognized toxic compounds. Our results raise concerns for recreation, harvesting of finfish and shellfish, and wildlife and desalination operations, highlighting the need for assessments and implementation of monitoring programs. Such programs appear to be particularly necessary in regions susceptible to urban influence.

## 1. Introduction

Cyanobacteria are ancient, ubiquitously distributed prokaryotes that have global biogeochemical and environmental significance in freshwater ecosystems. Some of these organisms are sources of bioactive metabolites that include recognized toxic compounds with human health significance [1,2]. Cyanobacteria are able to proliferate or bloom, sometimes to astonishingly high densities, in virtually all water types, although blooms show great variability in species composition, distribution, and magnitude. Bloom events have been reported at increasing frequency and intensity worldwide [3], and have been primarily attributed to trends of eutrophication, and warming of fresh waters associated with the changing climate [4,5,6,7]. Their ever-increasing prevalence is impacting and occasionally impairing the use of freshwater recreational water bodies, drinking water sources, and products designated for human consumption [8,9].

Cyanobacterial blooms can have a range of deleterious effects on water quality with possible far-reaching consequences. Concentrations of specific cyanotoxins in drinking water supplies, for instance, are now a priority concern of the World Health Organization (WHO) and the United States Environmental Protection Agency (US EPA) [10,11,12]. Humans may also encounter these chemicals by consuming contaminated finfish and shellfish, and via agricultural products irrigated with toxin-laden water [13,14,15,16]. Various cyanotoxins are also consequential to domestic animals, livestock, and wildlife, sometimes resulting in significant mortality events [15,17]. The best-studied and arguably most prevalent of these compounds are the cyclic heptapeptide microcystins, followed by nodularins [18,19,20]. Additional concerns include the small-molecule alkaloid saxitoxins, cylindrospermopsins, and anatoxins [1,21,22].

The associated effects of cyanobacterial blooms are not always immediate or acute, but can have a variety of longer-term/downstream consequences, including the disruption of normal ecosystem function [23]. For example, surface and sub-surface proliferations are able to decrease benthic vegetation by reducing light penetration through the water column. Additionally, the eventual senescence and sinking of decomposing cyanobacterial biomass can decrease dissolved oxygen concentrations, leading to hypoxia and a potential negative feedback cycle that favors the persistence of cyanobacteria [24].

In recent years, there has been increased recognition of various marine harmful algal blooms along the west coast of North America [25,26]. Despite heightened awareness of marine-sourced toxins in coastal ecosystems, and cyanotoxins in freshwater, the notion of cyanotoxin production within and/or transport into these coastal ecosystems has been largely ignored. Toxigenic cyanobacteria and/or cyanotoxins have been documented in a few estuaries in central California [27,28,29], but the extent and significance of their presence was not determined. Recent events and studies in the Monterey Bay National Marine Sanctuary, CA and the Puget Sound, WA highlight potentially widespread issues [30,31], but documentation of cyanobacterial community composition and associated toxins in brackish water/estuaries along the west coast of North America is still rudimentary, particularly in southern California.

Here we present the results of a discrete sample-based survey conducted in estuarine ecosystems and coastal lagoons along the Southern California Bight during September 2015. The recognition of harmful genera and occasionally toxins in streams [32], lakes/lentic water bodies [30,33], and rivers [33] that lead to the coast prompted this coordinated sampling effort at the land-sea interface. The objectives were to identify potential toxin-producing cyanobacterial taxa and to determine if specified cyanotoxins were present in detectable quantities within these brackish water ecosystems. We report the presence of multiple toxigenic species, and sometimes multiple cyanotoxins, at various locales in the Bight, raising the need for monitoring in these ecosystems.

## 2. Results

### 2.1. Santa Barbara/Ventura Counties

**Figure 1:**
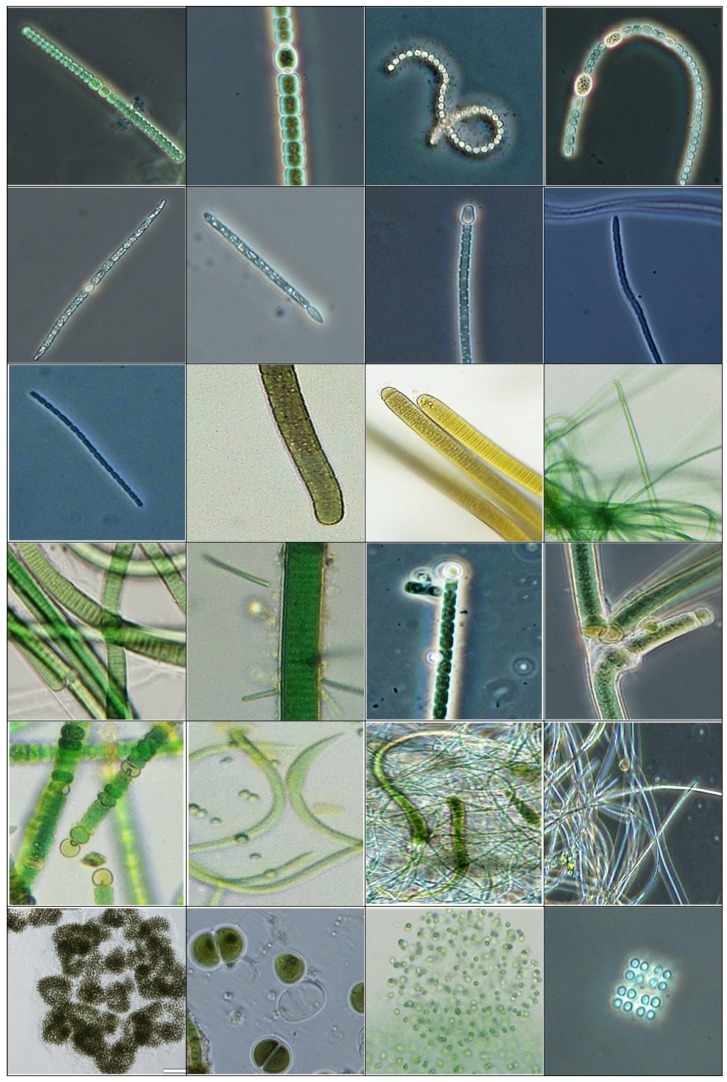
Photomicrograph (10–40x) collage of several cyanobacteria genera encountered in this study. **Top row**, from left: *Anabena* sp. 1, *Anabaena* sp. 2, *Dolichospermum* sp. 1, *Dolichospermum* sp. 2; **Second row**: *Aphanizonemon* sp., *Cylindrospermopsis* sp., *Cylindrospermum* sp., *Leptolyngbya* sp.; **Third row**: *Pseudanabaena* sp., *Phormidium* sp., *Oscillatoria* sp., *Geitlerinema* sp.; **Fourth row**: *Lyngbya* sp., *Heteroleiblenia* sp. on *Lyngbya* sp., *Nostoc* sp., *Tolypothrix* sp. Fifth row: *Nodularia* sp., *Planktolyngbya* sp., *Calotrhix* sp., *Leiblenia* sp.; **Bottom row**: *Microcystis* sp., *Chroococcus* sp., *Aphanocapsa* sp., *Merismopedia* sp.

Fifteen cyanobacterial genera were identified at twelve sites in Santa Barbara/Ventura County. *Aphanothece*, *Anabaena*, *Calothrix*, *Chroococcus*, *Cylindrospermum*, *Cylindrospermopsis*, *Dolichospermum*, *Lyngbya*, *Leptolyngbya*, *Merismopedia*, *Nodularia*, *Nostoc*, *Oscillatoria*, *Phormidium* and *Tolypothrix* (Table 1, Figure 1). The number of cyanobacterial genera observed at each sampling site ranged from one to nine in Santa Barbara/Ventura County. *Anabaena* (species *n* ≥ 3) was the most commonly observed genus (5 of 12 sites) and the Goleta Slough was the most diverse location in terms of species richness. Cyanotoxins were detected at eight of the twelve sampling sites (Table 2). Anatoxins were detected at Arroyo Hondo, Atascadero Creek, Santa Clara River Estuary, Tecolotito Creek, and the Goleta Slough using ultra high performance liquid chromatography with ultraviolet detection (HPLC-UV) with confirmation by ultra high performance liquid chromatography with fluorescence detection (HPLC-FL) (Agilent Technologies, Santa Clara, CA). Cylindrospermopsin was observed in the Santa Clara River and Calleguas Creek using HPLC-UV. Samples from Atascadero Creek, San Pedro Creek, and the Goleta Slough tested positive for microcystins by HPLC-UV, and at least one saxitoxin derivative was detected at Calleguas Creek and the Santa Clara River Estuary using ELISA (Table 2).

The Santa Clara River Estuary is a publically accessible nature preserve. This heavily impacted area receives approximately 8.5 million·gallons·day^−1^ of secondarily treated effluent from the Ventura Water Reclamation Facility. There is also an adjacent, active harbor, the Mandalay Generating Station to the south, intensive agriculture, and numerous golf courses in the region. Salinities at the time of sampling ranged from 3‰ to 8‰, and chl *a* concentrations were 24 to 56 µg·L^−1^. There was a nearly equal numerical ratio of diatoms to cyanobacteria in each of the plankton samples. Cyanobacterial genera included *Aphanothece*, *Anabaena*, *Chroococcus*, *Geitlerinema*, *Oscillatoria*, and *Phormidium* (Table 1). The presence of anatoxins and at least one saxitoxin derivative were confirmed (Table 2).

Calleguas Creek is part of a heavily impacted watershed affected from residential areas upstream, industry, and heavy agriculture that forms a drainage system to Mugu Lagoon. Salinities of multiple samples ranged from 1‰ to 4‰, and chl *a* was 35 to 43 µg·L^−1^. The potential toxin-producing cyanobacteria were *Cylindrospermopsis*, *Dolichospermum*, and *Nostoc* (present at relatively high abundances). Cylindrospermopsin and at least one saxitoxin derivative were detected in Calleguas Creek (Table 2).

The Goleta Slough is surrounded by an airport, a university, and a heavily populated residential area. This system is also influenced by local agriculture and nutrient concentrations have historically reflected this activity. The potential toxin producing cyanobacteria present were *Anabaena*, *Calothrix*, *Cylindrospermum*, *Dolichospermum*, *Leptolyngbya*, *Merismopedia*, *Oscillatoria*, and *Phormidium* (Table 1). Salinities of samples at this location ranged from 1‰ to 34‰ and chl *a* ranged from 18 to ~40 µg·L^−1^. The presence of anatoxin-a and microcystins were confirmed in the Goleta Slough (Table 2).

### 2.2. Los Angeles County

Seventeen genera of cyanobacteria were identified from sixteen sites in coastal Los Angeles County. *Aphanizonemon*, *Aphanocapsa*, *Aphanothece*, *Anabaena*, *Calothrix*, *Chroococcus*, *Geitlerinema*, *Gloeocapsa*, *Heteroleibleinia*, *Leibleinia*, *Lyngbya*, *Leptolyngbya*, *Merismopedia*, *Microcystis*, *Nodularia*, *Oscillatoria*, and *Phormidium* were present (Table 1, Figure 1), with *Chroococcus* and *Oscillatoria* being the most commonly observed (5 of 16 sites). The number of cyanobacterial genera observed at each sampling site ranged from zero to nine. Cyanotoxins were detected at five localities in Los Angeles County (Table 2). Anatoxins were detected in Malibu Lagoon and Topanga Creek using HPLC-UV with further confirmation by HPLC-FL. Microcystins, identified with HPLC-UV, were found in Topanga Creek, Topanga Lagoon, and Ballona Creek. At least one saxitoxin derivative was detected in Ballona Lagoon by ELISA (Table 2).

The Ballona Lagoon is bordered by heavy development, including a residential area and active recreational harbor. The cyanobacterial genera *Aphanizomenon*, *Chroococcus*, and *Leptolyngbya* were present in the lagoon. Saxitoxin(s) were detected, but were likely attributed to the relatively high abundance of the marine dinoflagellate *Alexandrium catenella* in the area (Table 2). Another dinoflagellate, *Akashiwo sanguinea*, and a mixture of centric diatoms were also present at high relative abundances. Chl *a* concentration in the sample was 28 µg·L^−1^ and salinity was 30‰. 

Topanga Lagoon is influenced by light development upstream and is separated from the ocean by a berm during most of the year due to low, and highly seasonal, regional precipitation rates. This watershed is heavily influenced by seasonal rains. Topanga was the most diverse lagoonal site in terms of co-occurring cyanobacterial genera within Los Angeles County in both planktonic and benthic samples. Organisms of interest included *Chroococcus*, *Geitlerinema*, *Gloeocapsa*, *Lyngbya*, *Oscillatoria* spp., and *Phormidium* spp., plus a mixture of diatoms. The salinity ranged from 20‰ to 24‰ and chl *a* varied between 13 and 18 µg·L^−1^. Microcystins were detected in benthic samples from Topanga Lagoon (Table 2).

### 2.3. Orange County

Nine cyanobacterial genera were identified at nine sampling locations within Orange County, including *Aphanocapsa*, *Aphanothece*, *Calothrix*, *Chroococcus*, *Leibleinia*, *Leptolyngbya*, *Merismopedia*, *Oscillatoria*, and *Phormidium* (Table 1; Figure 1). The most commonly observed cyanobacteria were *Leibleinia* and *Leptolyngbya* (4 of 9 sites). Cyanotoxins were detected at two of eight sites; Newport Back Bay and San Juan Creek. Microcystins were found in Back Bay using HPLC-UV and saxitoxin(s) in San Juan Creek by ELISA. Anatoxins were also detected at San Juan Creek by screening with HPLC-UV and confirmed using HPLC-FL (Table 2).

Newport Back Bay is a densely populated residential area that receives runoff from the surrounding community as well as influence from boating and other recreational activities. This sampling location was the most diverse in terms of co-occurring genera in all of Orange County with *Aphanocapsa*, *Lyngbya*, *Leptolyngbya*, *Merismopedia*, and *Oscillatoria* present. A water sample with 15 µg·L^−1^ chl *a* and salinity of 6‰ contained at least two microcystin derivatives (Table 2).

San Juan Creek enters the Pacific Ocean at Doheny State Beach Lagoon. This watershed is moderately developed and receives runoff from agriculture and other point-sources. Potentially toxic cyanobacteria included *Aphanocapsa* and three species of *Oscillatoria* that were not observed at other sampling sites in Orange County. A benthic sample from San Juan Creek tested positive for anatoxins and at least one saxitoxin derivative (Table 2), while toxins were not detected in the plankton sample from that location. The salinity was 8‰ and no chl *a* measurements on benthic samples were obtained. The corresponding sub-surface water sample had a chl *a* concentration of 12 µg·L^−1^.

### 2.4. San Diego County

Twenty one genera were identified from fifteen sites in San Diego County. These taxa were *Aphanocapsa*, *Aphanothece*, *Anabaena*, *Calothrix*, *Chroococcus*, *Cylindrospermum*, *Cylindrospermopsis*, *Dolichospermum*, *Geitlerinema*, *Gloeocapsa*, *Leibleinia*, *Lyngbya*, *Leptolyngbya*, *Merismopedia*, *Microcystis*, *Nodularia*, *Nostoc*, *Oscillatoria*, *Phormidium*, *Planktolyngbya*, and *Tolypothrix* (Table 1, Figure 1). The number of cyanobacterial genera observed at each sampling site ranged from 0‰ to 6‰. The most commonly observed genera were *Anabaena*, *Leptolyngbya*, and *Oscillatoria* (5 of 15 sites) and the most diverse sampling sites in terms of co-occurring cyanobacterial genera were Loma Alta Creek, Batiquitos Lagoon, San Diego Creek, and the Tijuana River Estuary. Cyanotoxins were detected at seven of fifteen sites in San Diego County (Table 2). Anatoxins were identified in samples from the Santa Margarita River, Buena Vista, and Batiquitos Lagoons using HPLC-UV and HPLC-FL. HPLC-UV was used to identify cylindrospermopsin in the lower salinity reaches of Buena Vista Lagoon as well as microcystins in samples from the Santa Margarita River, Buena Vista Lagoon, Batiquitos Lagoon, San Elijo Lagoon, Loma Alta Creek, Sweetwater River, and the Tijuana River Estuary. Nodularin was identified by HPLC-UV solely in the Santa Margarita River (Table 2). Saxitoxin(s) were detected in the Santa Margarita River, Batiquitos Lagoon, San Elijo Lagoon, Loma Alta Creek, and the Sweetwater River using ELISA (Table 2).

The Buena Vista creek and lagoon system is surrounded by a densely populated residential zone, has several small-scale inputs, and rarely exchanges water with the adjacent Pacific Ocean. This location was sampled in multiple locations including both sub-surface water and benthic grab samples. Potential toxin-producing cyanobacteria included *Cylindrospermopsis*, *Microcystis*, and two *Oscillatoria* spp. Anatoxins, cylindrospermopsin, and microcystins were detected (Table 2). The salinities of samples collected in the area ranged from 2‰ to 30‰, and chl *a* concentrations of water samples varied from 8 to 24 µg·L^−1^.

The watershed connecting to Loma Alta Creek is heavily developed, including industry, agriculture, and numerous golf courses. This area was also sampled in multiple locations, including sub-surface water and the benthos. A range of cyanobacteria were identified; *Aphanizomenon*, *Chroococcus*, *Cylindrospermum*, *Leptolyngbya*, *Microcystis*, and *Phormidium*. Microcystins and the presence of at least one saxitoxin derivative were detected in the same benthic sample (Table 2). The salinity among the water samples varied from 1‰ to 23‰, and chl *a* ranged from 20 to ~45 µg·L^−1^

Overall, across the entire Southern California Bight, there were twelve sites where two or more cyanotoxins were detected in the same sample (23% of sites). Anatoxins were identified at 11 sites (21%), cylindrospermopsin (3 sites, 6%), microcystins (14 sites; 26%), nodularin (1 site; 2%), and saxitoxin(s) (7 sites; 13%) (Table 2).

## 3. Discussion

The prevalence, geographical distribution, health implications, and ecological impacts of cyanobacteria and their associated toxins within estuarine environments and along marine coastlines are very poorly characterized. Our lack of awareness of the potential importance of these aspects for ecosystem health has been underscored by recent mortality events among sea otters in the aforementioned Monterey Bay National Marine Sanctuary, whose cause was eventually attributed to the transfer of freshwater-derived microcystins into marine food webs at the river-sea interface in central California [30,34,35]. Subsequent studies in the northwest United States and Japan have identified contamination of marine resources from inland/freshwater sources of microcystins [31,36].

Microcystins are not the only cyanotoxins of concern in coastal waters. Nodularins were documented decades ago in food webs of the Baltic Sea [37,38], Oceania [39], Greece, and Portugal [40]. To our knowledge, nodularin was documented for the first time in California during 2015 ([41], this study). Threats to seafood safety from cyanotoxins other than microcystins in brackish waters have also been reported in various parts of the world [42]. At present, marine environments and related consumables are rarely screened for these compounds unless they occur in an area that is known to be at risk. The seminal studies noted above have begun to draw attention to the possibility of cyanobacterial toxins as unanticipated stressors, and potentially multiple stressors when considered together with toxins of marine origin, for organisms living at or near the intersection of marine and freshwater ecosystems.

Our lack of recognition regarding the importance of algal and cyanobacterial blooms and their toxins at these interface ecosystems has been hindered, in part, by distinct fields of study that tend to focus somewhat exclusively on freshwater or marine ecosystems (limnology and oceanography), reinforced by funding compartmentalization which generally funds work on harmful blooms in either domain, but not both. Investigations at the intersection of these environments therefore have been rare. Moreover, research on phytoplankton toxins in Southern California has largely focused on marine algae because of increased awareness resulting from visible manifestations of these blooms, and their correlation with wildlife mass mortality events for nearly two decades [43,44]. In contrast, the widespread distribution of potentially toxic cyanobacteria in freshwater ecosystems across the region has been recognized only recently [32]. As a consequence, scientific recognition and understanding of the importance of cyanotoxins of freshwater origin in estuaries throughout the Southern California Bight (and an unknown number of other regions globally) has significantly lagged behind our knowledge of marine systems.

Here we present new information on the extensive occurrence of potentially toxic cyanobacterial genera, as well as the confirmation of several groups of cyanotoxins, in brackish/marine influenced waters along the coast of the Southern California Bight. We surveyed all accessible brackish waters along the Bight coastline during late summer/early fall because average annual precipitation is minimal at that time and temperature is maximal (conditions which tend to favor proliferation and accumulations of cyanobacteria). We found that potential producers of cyanotoxins existed at high abundances at all sites, and many taxa exhibited wider distributions than previously documented or anticipated. Multiple genera were present at most sites (range of 0–9, average of 3 genera at each site). Collectively, we identified twenty-three of approximately forty known toxic genera of cyanobacteria. We also detected each of the cyanotoxins investigated and report at least two groups of compounds at twelve sites (23% of all sites). Our findings are based solely on material from discrete water samples collected on filters, rather than dissolved toxins detected using passive, integrative sampling methods such as Solid Phase Adsorption Toxin Tracking [45,46]. Thus, we speculate that our approach yielded a lower limit estimate of the number of sites at which toxins were present. Additionally, we may have overlooked some compounds due to low concentrations present in the samples or due to the sensitivity of the UV detector. Nonetheless, this study provides a baseline for future investigations of temporal and spatial distributions of cyanotoxins along the margin of the Southern California Bight.

The specific source(s) of the toxins observed in this study (i.e., cyanobacterial genera responsible for the toxins) are difficult to assess because of the potential that several of the genera observed are capable of producing toxins, and toxigenic species do not always produce toxins. Additionally, different populations of the same taxon may biosynthesize different compounds due to site-specific environmental drivers and genetic composition [47], and toxic and non-toxic strains can occur within a species [48,49]. Therefore, the same species may produce toxin(s) in one environment but not another, and specific classes of toxins may be produced by multiple organisms in a given area [50]. In the current study, for example, microcystins were detected at fourteen sites (26%) while *Microcystis* (a major producer of microcystins) was only identified at four (of which 3 sites, 75%, tested positive), indicating that other taxa were producing microcystins at some of our study sites. Elucidating the specific producers of the cyanotoxins observed at our study sites, their physiological responses to environmental conditions, will allow better understanding of the factors leading to the occurrence of toxins at the land-sea interface.

In addition to strain/species-related variance in toxin production as noted above, cyanobacterial abundances and toxin concentrations exhibit considerable spatial heterogeneity in natural systems due (at least in part) to substantial small-scale variations in environmental conditions [51]. The difference in strain prevalence is partially dictated by physiological response to abiotic factors [52,53]. Most estuaries/brackish waters inherently have complex gradients of abiotic factors, and thus are candidate habitats for finding multiple co-occurring cyanobacteria and associated toxins, a generalization consistent with our findings. Heterogeneity can be pronounced within these systems in the plankton and the benthos. Spatial heterogeneity of cyanobacteria and associated toxins has been reported from lake systems [51,54] and was also evident in the current study. Several of our sites had pronounced differences in vertical and horizontal community structure and cyanotoxins (unpublished), which were partially attributed to salinity. Examples include the Buena Vista Lagoon, the Goleta Slough, the Santa Clara River Estuary, and Loma Alta Creek. Buena Vista, in particular, was dominated by *Cylindrospermopsis* at salinities of 2‰–3‰, *Microcystis* at salinities of ~6‰–10‰, and *Oscillatoria* at salinities up to 30‰. Cylindrospermopsin was present in a sample with a salinity of 2‰, while at 6‰, microcystins and anatoxins were also detected. Microcystins were the only compounds identified in samples above 10‰. Heterogeneity undoubtedly plays a role in creating highly localized and possibly ephemeral hotspots with elevated toxin concentrations, and complicates the task of obtaining accurate assessments of toxins in estuarine environments.

We anticipated that sites in our study adjacent to or within urbanized areas might demonstrate greater cyanobacterial diversity or the presence of multiple toxins compared to less developed locations because of increased nutrient loading contributed by developed areas. In general, cyanobacterial proliferations in fresh water and harmful algal blooms in coastal ecosystems appear to be increasing with the alteration of water quality parameters affected by human population expansion, large scale droughts, and other climate induced shifts [6,55,56]. Factors such as temperature, nutrients (concentrations, speciation and ratios), and conductivity undoubtedly influence the growth, biomass, toxin production, and types of cyanobacteria present (succession patterns) [52]. However, a clear relationship between toxin occurrence and drainage from highly urbanized sites was not evident in our survey. We observed relatively similar amounts of biomass and diversity across highly urbanized versus less-urbanized areas, although highly influenced sites such as the Santa Clara River Estuary, Marina del Rey, Ballona Lagoon, and the San Gabriel and Tijuana Rivers did have higher relative abundances of diatoms and dinoflagellates compared to (relative to) cyanobacteria. Overall, few positive samples were obtained in southern Los Angeles County and Orange County, arguably the most urbanized areas in our study region. We speculate that the high percentage of impervious surfaces in urbanized areas, highly channelized water movement, and possibly anthropogenic substances that might adversely affect cyanobacterial growth and accumulation may have limited bloom development. These factors may explain the lack of correlation between urbanization and cyanobacterial toxins.

Multiple cyanotoxins in discrete water samples were detected at several locations in the present study (12 sites; 23%). Such co-occurrences of cyanotoxins have recently been reported in ponds/lakes [57,58,59,60], drinking water reservoirs [61,62], rivers [63], and brackish waters [64]. These mixtures, consisting of chemicals that often exhibit different modes of biochemical action, are sources of contamination with potentially important but currently poorly defined health implications. Combined with the potential for toxins of marine origin, these biotoxins may act in an additive or synergistic manner, as multiple physiological stressors for organisms living at the land-sea interface.

We have documented a previously undocumented environmental and public health concern relating to cyanobacterial toxins along the coastline of the Southern California Bight. Our results provide a clearer recognition of the connections between freshwater, estuarine, and marine habitats with respect to our understanding of cyanobacteria and their toxins. Future studies documenting seasonality and interannual variability in toxin presence, spatial heterogeneity within these ecosystems, quantitative data on multiple toxins, and the identity of toxin producers will enable the implementation of surveillance and monitoring efforts for cyanobacteria and cyanotoxins at the land-sea interface in Southern California waters.

## 4. Methods

### 4.1. Sample Collection and Sites

**Figure 2:**
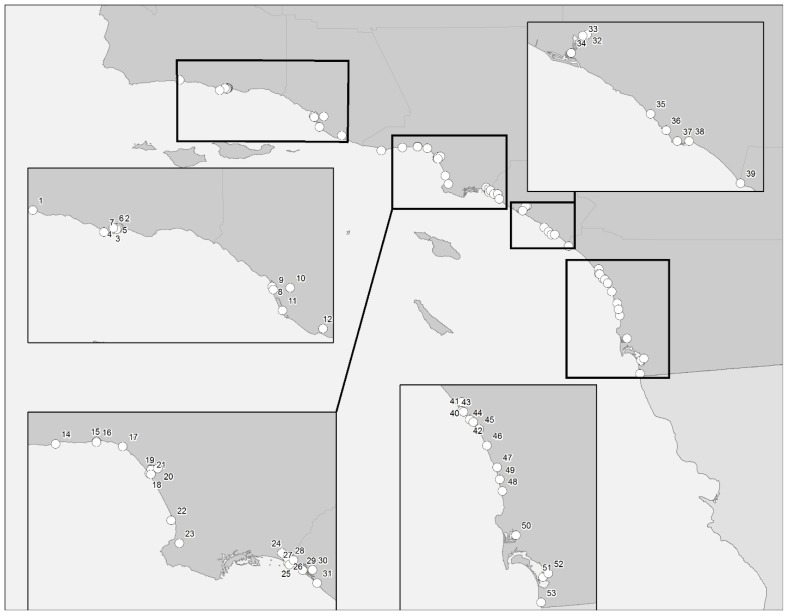
Map depicting fifty-three sampling locations along the Southern California Bight coastline. Santa Barbara and Ventura Counties (#1–12) 1 = Arroyo Hondo, 2 = Atascadero Creek, 3 = San Jose Creek, 4 = San Pedro Creek, 5 = Tecolotito Creek, 6 = Goleta Slough, 7 = Devereaux Slough, 8 = Ventura Harbor, 9 = Santa Clara River Estuary, 10 = Santa Clara River, 11 = Channel Islands Harbor, 12 = Calleguas Creek. Los Angeles County (#13–28), 13 = Zuma Lagoon, 14 = Malibu Lagoon, 15 = Topanga Creek, 16 = Topanga Lagoon, 17 = Rustic Creek, 18 = Ballona Lagoon, 19 = Marina Del Rey, 20 = Ballona Creek, 21 = Del Rey Lagoon, 22 = King Harbor, 23 = Malaga Creek, 24 = Colorado Lagoon, 25 = Alamitos Bay, 26 = Mother’s Beach, 27 = San Gabriel River, 28 = San Gabriel River upstream. Orange County (#29–39), 29 = Seal Beach, 30 = Huntington Harbour, 31 = Bolsa Chica Channel/Basin, 32 = San Diego Creek, 33 = Upper Newport, 34 = Back Bay, 35 = Aliso Creek, 36 = Salt Creek, 37 = Dana Point Harbor, 38 = San Juan Creek, 39 = San Mateo Creek. San Diego County (#40–53), 40 = Santa Margarita River, 41 = Oceanside Harbor, 42 = Loma Alta Creek, 43 = San Luis Rey River, 44 = Buena Vista Creek/Lagoon, 45 = Agua Hendionda, 46 = Batiquitos Lagoon, 47 = San Elijo Lagoon, 48 = Los Penasquitos, 49 = San Dieguito River, 50 = Mission Bay, 51 = San Diego Bay, 52 = Sweetwater River, 53 = Tijuana River/Estuary.

A survey was conducted during the third week of September 2015 to begin assessing cyanotoxins and toxin producers in coastal ecosystems along the Southern California Bight. Samples were collected from sub-surface and benthic locations at each site when possible. At least 500 mL were collected for water samples, while grabs of visible mats/substrate were collected for benthic samples and placed into whirl-pak™ bags. More than one sample was obtained at each site when multiple features were visibly present. All samples were placed into coolers and returned to the lab within twenty four hours of collection. A total of 53 sites (Figure 2) are listed with GPS coordinates in Table S1.

### 4.2. Cyanobacterial Taxonomy

Potential toxin-producing cyanobacteria were identified to the genus level according to Komárek [65,66,67,68,69,70,71]. Briefly, samples were homogenized by successive inversions and an aliquot was poured into 20 mL tissue culture dishes, settled overnight, and viewed under an Olympus CKX41 inverted microscope (Olympus America, Center Valley, PA, USA)

Chlorophyll *a* analysis and salinity (ancillary measurements).

Samples for chlorophyll *a* (Chl *a*) measurements were filtered in duplicate onto 25 mm GF/F filters (GE Whatman, Marlborough, MA, USA). 5 ml of 90% acetone was added and each vial was allowed to extract for twenty-four hours at −20 °C in the dark. Chl *a* was determined using a Turner Designs 10-AU fluorometer (Turner Designs, Sunnyvale, CA, USA) [72]. Salinity was measured with a handheld refractometer.

### 4.3. Cyanotoxin Screening/Assessment

Cyanotoxins were not quantified in this survey. The specific goals of this study were to determine what types of cyanotoxins were present and locate sites with multiple toxins in the region. Particulate material was collected on 47 mm GF/F filters to obtain sufficient cyanobacterial biomass from sub-surface water samples. The filters were subjected to three freeze-thaw cycles in a minimal amount of 10% aqueous methanol. After the third cycle, the volume was adjusted to 1 mL with 50% aqueous methanol and agitated to a slurry with a glass rod. The samples were filtered into multiple 1.5 mL amber glass HPLC vials using a glass luer-lock syringe and 13 mm 0.22 µm PFTE filters. Subsamples diluted with nanopure water or 50% aqueous methanol were used for analysis and the remainder stored at −20 °C. A duplicate 25 mm GF/F filter was taken for saxitoxin analysis and extracted with 10% aqueous methanol.

Cyanotoxin analysis was primarily conducted using ultra high performance liquid chromatography with HPLC-UV using an Agilent Infinity 1260. Separation was carried out with an Agilent Zorbax RRHD Eclipse Plus C18, 2.1 × 50 mm, 1.8 µm column (Agilent Technologies, Santa Clara, CA, USA). The method was adapted and modified from [57,73,74]. The mobile phase consisted of A: H_2_0, 0.01% TFA, B: MeCN, 0.01% TFA. The elution gradient began with 20% B from zero to 4 min, with a transition to 70% B by 4.2 min. This was followed by an increase to 95% B by 4.6 min and this was held until 5.4 min, with a subsequent decrease to 20% B by 6 min, and held for 1.5 min. The flow rate was 1 mL/min and the column was heated to 30 °C. Anatoxin-a and derivatives were considered ‘anatoxin positive’ due to lack of ion fragmentation patterns obtained from HPLC-UV analysis. Any putative anatoxin positives were followed with more specific methodology using the same HPLC that involves derivatization with n-BFD and fluorescence detection [75,76].

Particulate domoic acid was also analyzed in samples that included the diatoms *Nitzschia* and/or *Pseudo-nitzschia* by HPLC-UV using a Phenomenex C-18 luna column (Phenomenex, Torrance, CA, USA) with the methodology described in [77]. Screening for saxitoxins was performed using a MAX Signal ELISA kit from Bioo Scientific (Bioo Scientific, Austin, TX, USA). Compounds were identified based on retention times and absorbance spectra facilitated by certified reference standards obtained from the National Resource Council of Canada. Additionally, phenylalanine was purchased from Sigma Aldrich (Sigma Aldrich, St. Louis, MO, USA)

## Figures and Tables

**Table 1:** Relative abundances of cyanobacteria genera across fifty-three sampling locations along the Southern California Bight coastline. Dominant = D (>50%), Abundant = A (25%–49%), Common = C (10%–24%), Present = P (1%–9%), Rare = R (<1%). Multiple letters per box denotes multiple species of a given genera per site.

Locality	*Aphanizomenon*	*Aphanocapsa*	*Aphanothece*	*Anabaena*	*Calothrix*	*Chroococcus*	*Cylindrospermum*	*Cylindrospermopsis*	*Dolichospermum*	*Geitlerinema*	*Gloeocapsa*	*Heteroleibleinia*	*Leibleinia*	*Lyngbya*	*Leptolyngbya*	*Merismopedia*	*Microcystis*	*Nodularia*	*Nostoc*	*Oscillatoria*	*Phormidium*	*Planktolyngbya*	*Topolythrix*
Arroyo Hondo														C	A					AC	A		
Atascadero Creek				AC	C													P					
San Jose Creek							D		R														
San Pedro Creek																P		A			A		
Tecolotito Creek																			D				
Goleta Slough				A	C		R		R						P	P				C	R		P
Devereaux Slough														D									
Ventura Harbor			P											A						A			
Santa Clara River Estuary			P	C		A				P										A	R		
Santa Clara River				PP				P	P										A				
Channel Islands Harbor					D																		
Calleguas Creek								A	A										A				
Zuma Lagoon					P										D	A							
Malibu Lagoon				PPR														P		A			
Topanga Creek			P			P				P	P	P			P	P				CC	C		
Topanga Lagoon						A					P	P		P	C	P				DP			
Rustic Creek																							
Ballona Lagoon	R					D									R								
Marina Del Rey												R	P							D			
Ballona Creek	C			P													D						
Del Rey Lagoon			P			D						P	P	R									
King Harbor																							
Malaga Creek		P								D						A							
Colorado Lagoon																							
Alamitos Bay																							
Mother's Beach						D														A			
San Gabriel																							
San Gabriel up																							
Seal Beach																							
Huntington Harbour													D										
Bolsa Chica Channel													D										
Upper Newport		D											P		A								
Back Bay		D													C	A				P			
Aliso Creek			P			D							P								P		
Salt Creek																							
Dana Point Harbor					D																		
San Juan Creek		P																		DAC			
San Mateo Creek																							
Santa Margarita				P			A						R		R			A		P			
Oceanside Harbor																							
San Luis Rey River								A									D						
Buena Vista Creek/Lagoon								D									A			CC			
Agua Hendionda														D									
Batiquitos Lagoon				A							C				C			A	P	P			
San Elijo Lagoon						A														D		P	
Los Penasquitos Lagoon					P									D									
San Dieguito River		D																					
San Diego Creek				A		R							R		P	C				D			
Loma Alta Creek	R					P	P								A		P				A		
Mission Bay					P									C	D								P
San Diego Bay										C					D								
Tijuana River/Estuary				A		A			P		P					P							
Sweetwater River				R												P		P		A	A		

**Table 2:** Cyanotoxin prevalence across fifty-three sampling locations along the Southern California Bight coastline.

Locality	Anatoxins	Cylindro-Spermopsins	Microcystins	Saxitoxins	Nodularins
Arroyo Hondo	+	−	−	−	−
Atascadero Creek	+	−	+	−	−
San Jose Creek	−	−	−	−	−
San Pedro Creek	−	−	−	−	−
Tecolito Creek	+	−	−	−	−
Goleta Slough	+	−	+	−	−
Devereaux Slough	−	−	−	−	−
Ventura Harbor	−	−	−	−	−
Santa Clara River Est/Lagoon	+	−	−	+	−
Santa Clara River	−	+	+	−	−
Channel Islands Harbor	−	−	−	−	−
Calleguas Creek	−	+	−	+	−
Zuma Lagoon	−	−	−	−	−
Malibu Lagoon	+	−	−	−	−
Topanga Creek	+	−	+	−	−
Topanga Lagoon	−	−	+	−	−
Rustic Creek	−	−	−	−	−
Ballona Lagoon	−	−	−	+	−
Marina Del Rey	−	−	−	−	−
Ballona Creek	−	−	+	−	−
Del Rey Lagoon	−	−	−	−	−
King Harbor	−	−	−	−	−
Malaga Creek	−	−	−	−	−
Colorado Lagoon	−	−	−	−	−
Alamitos Bay	−	−	−	−	−
Mother's Beach	−	−	−	−	−
San Gabriel River	−	−	−	−	−
San Gabriel River upstream	−	−	−	−	−
Seal Beach	−	−	−	−	−
Huntington Harbour	−	−	−	−	−
Bolsa Chica Channel/Basin	−	−	−	−	−
Upper Newport	−	−	−	−	−
Back Bay	−	−	+	−	−
Aliso Creek	−	−	−	−	−
Salt Creek	−	−	−	−	−
Dana Point Harbor	−	−	−	−	−
San Juan Creek	+	−	−	+	−
San Mateo Creek	−	−	−	−	−
Santa Margarita	+	−	+	+	+
Oceanside Harbor	−	−	−	−	−
San Luis Rey River	−	−	−	−	−
Buena Vista Creek/Lagoon	+	+	+	−	−
Agua Hendionda	−	−	−	−	−
Batiquitos Lagoon	+	−	+	+	−
San Elijo Lagoon	−	−	+	+	−
Los Penasquitos	−	−	−	−	−
San Dieguito River	−	−	−	−	−
San Diego Creek	−	−	−	−	−
Loma Alta Creek	−	−	+	+	−
Mission Bay	−	−	−	−	−
San Diego Bay	−	−	−	−	−
Tijuana River/Estuary	−	−	+	−	−
Sweetwater River	−	−	+	+	−

## Supplementary Materials

The following are available online at www.mdpi.com/2072-6651/9/3/95/s1, Table S1: List of sampling locations along the Southern California Bight coastline.
