# Supplementary material for: Multiple Stressors at the Land-Sea Interface: Cyanotoxins at the Land-Sea Interface in the Southern California Bight
Source: Toxins (Basel). 2017 Mar 9;9(3):95. doi: 10.3390/toxins9030095 (PMC5371850; doi:10.3390/toxins9030095)
Supplement: Supplementary file 1 [file toxins-09-00095-s001.pdf]

Supplementary Materials: Multiple Stressors at the Land-Sea Interface: Cyanotoxins at the Land-Sea Interface in the Southern California Bight

Avery O. Tatters, Meredith D.A. Howard, Carey Nagoda, Lilian Busse, Alyssa G. Gellene and David A. Caron

Table 1. List of sampling locations along the Southern California Bight coastline.

Site no.	Collection site	Latitude	Longitude
1	Arroyo Hondo	34.475887	−120.142374
2	Atascadero Creek	34.422523	−119.823780
3	San Jose Creek	34.424082	−119.827486
4	San Pedro Creek	34.421232	−119.830163
5	Tecolotito Creek	34.418000	−119.833659
6	Goleta Slough	34.422102	−119.845172
7	Devereaux Slough	34.409697	−119.880109
8	Ventura Harbor	34.241000	−119.264000
9	Santa Clara River Estuary	34.232180	−119.260396
10	Santa Clara River	34.237680	−119.197751
11	Channel Islands Harbor	34.169000	−119.227000
12	Calleguas Creek	34.112213	−119.079411
13	Zuma Lagoon	34.014135	−118.820756
14	Malibu Lagoon	34.033760	−118.682106
15	Topanga Creek	34.040031	−118.583041
16	Topanga Lagoon	34.038549	−118.583066
17	Rustic Creek	34.027905	−118.519305
18	Ballona Lagoon	33.962537	−118.454495
19	Marina Del Rey	33.971706	−118.450240
20	Ballona Creek	33.974475	−118.433834
21	Del Rey Lagoon	33.960382	−118.450926
22	King Harbor	33.848949	−118.401446
23	Malaga Creek	33.794100	−118.381934
24	Colorado Lagoon	33.770557	−118.133009
25	Alamitos Bay	33.747390	−118.118825
26	Mother's Beach	33.758569	−118.119577
27	San Gabriel River	33.742407	−118.114911
28	San Gabriel River upstream	33.752656	−118.104856
29	Seal Beach	33.728441	−118.082656
30	Huntington Harbour	33.728907	−118.058610
31	Bolsa Chica Channel/Basin	33.697001	−118.047653
32	San Diego Creek	33.651163	−117.866067
33	Upper Newport	33.649684	−117.873630
34	Back Bay	33.619043	−117.894136
35	Aliso Creek	33.510813	−117.752799
36	Salt Creek	33.481601	−117.724729
37	Dana Point Harbor	33.462161	−117.705311
38	San Juan Creek	33.462203	−117.684102
39	San Mateo Creek	33.387295	−117.592901
40	Santa Margarita River	33.237637	−117.396464
41	Oceanside Harbor	33.207396	−117.395188
42	Loma Alta Creek	33.177303	−117.368736
43	San Luis Rey River	33.203937	−117.389797
44	Buena Vista Creek/Lagoon	33.168145	−118.826982
45	Agua Hendionda	33.144478	−117.336636
46	Batiquitos Lagoon	33.088702	−117.309584
47	San Elijo Lagoon	33.013767	−117.275189
48	Los Penasquitos	32.932840	−117.257526
49	San Dieguito River	32.972259	−117.266138
50	Mission Bay	32.782587	−117.210519
51	San Diego Bay	32.639170	−117.117971
52	Sweetwater River	32.652057	−117.099748
53	Tijuana River/Estuary	32.552610	−117.124614
